# Higher-Order Conditioning: What Is Learnt and How it Is Expressed

**DOI:** 10.3389/fnbeh.2021.726218

**Published:** 2021-09-09

**Authors:** Robert C. Honey, Dominic M. Dwyer

**Affiliations:** School of Psychology, Cardiff University, Cardiff, United Kingdom

**Keywords:** association, behavior, Pavlovian conditioning, similarity, timing

## Abstract

Pairing a neutral conditioned stimulus (CS) with a motivationally significant unconditioned stimulus (US) results in the CS coming to elicit conditioned responses (CRs). The widespread significance and translational value of Pavlovian conditioning are increased by the fact that pairing two neutral CSs (A and X) enables conditioning with X to affect behavior to A. There are two traditional informal accounts of such higher-order conditioning, which build on more formal associative analyses of Pavlovian conditioning. But, higher-order conditioning and Pavlovian conditioning have characteristics that are beyond these accounts: Notably, the two are influenced in different ways by the same experimental manipulations, and both generate conditioned responses that do not reflect the US *per se*. Here, we present a formal analysis that sought to address these characteristics.

## Introduction

Pavlov observed that dogs given pairings of light with food came to salivate during the light, but also during a tone that was later paired with the light. In his terms, the light (a conditioned stimulus, CS) had become a substitute for food (an unconditioned stimulus, US), evidenced both through the capacity of the light to elicit salivation (the conditioned response, CR) and to support a “*reflex of the second order*” to the tone. In fact, Pavlov described such second-order CRs as “*in most cases very weak*,” indicating that there were substantial individual differences in their size and transience (Pavlov, 1927; pp. 104–105). We will return to the important issue of individual differences towards the end of this article. For now, it is sufficient to note that second-order conditioning is a well-established phenomenon across a range of preparations (e.g., *appetitive conditioning*: Rashotte et al., 1977; *aversive conditioning*: Rizley and Rescorla, 1972; *sexual conditioning*: Crawford and Domjan, 1995), and so too is another example of higher-order conditioning, sensory preconditioning (e.g., *appetitive conditioning*: Allman and Honey, 2006; *aversive conditioning*: Brogden, 1939; *flavor-aversion learning*: Rescorla and Cunningham, 1978). For sensory preconditioning, the tone and light in the opening example are paired before the light is conditioned, whereupon the tone also elicits conditioned responding (see Table 1).

**Table 1:** Higher-order conditioning procedures.

	Stage 1	Stage 2	Test
Second-order conditioning:	X→US	A→X	A?
Sensory preconditioning:	A→X	X→US	A?

*Note: A and X are conditioned stimuli and the US denotes an unconditioned stimulus*.

Higher-order conditioning procedures have become a popular means of examining the neurobiology of learning and memory (for a review, see Gewirtz and Davis, 2000; see also, e.g., Lin and Honey, 2011; Gilboa et al., 2014; Holland, 2016; Lin et al., 2016; Lay et al., 2018; Maes et al., 2020; Mollick et al., 2020). This popularity reflects the relevance of higher-order conditioning to clinical domains (e.g., Davey and Arulampalan, 1982; Davey and McKenna, 1983; Wessa and Flor, 2007; see also, Field, 2006; Haselgrove and Hogarth, 2011), but also the practical advantages of the procedures, and the potential insights that their use enables: The procedures allow the complex effects generated by the presentation of a motivationally significant US, on X→US trials, to be separated from the associative processes operating on A→X trials; and they also allow the nature of different acquisition and performance processes to be separately probed. But, what is learned during higher-order conditioning and how is that learning expressed? These two related questions have not been addressed in an integrated fashion by traditional accounts of higher-order conditioning. In fact, a recent critical review of evidence relating to these accounts suggested that they leave many important issues unresolved, which motivated the development of a new computational model of higher-order conditioning (Honey and Dwyer, under review). This model was built on a recent analysis of Pavlovian conditioning and performance: HeiDI (Honey et al., 2020a). Here, we first present a synthesis of extant informal accounts of higher-order conditioning together with the evidence that they fail to address, before presenting the new computational model of higher-order conditioning.

## Traditional Accounts of Higher-Order Conditioning

**Figure 1:**
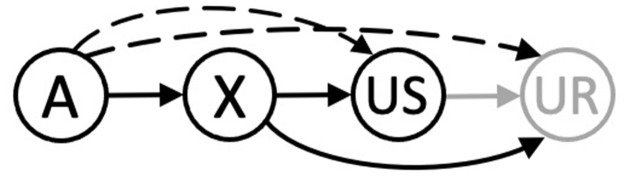
An integrated schematic for higher-order conditioning: The associative chains (black continuous arrows) and retrieval mediated associations (black dashed arrows) resulting from separate A→X and X→US trials. The gray solid arrow is an (unconditioned) link between the US its UR. The A, X, and US nodes are held to be activated by their corresponding stimuli, with the UR generated by the presentation of the US.

Mackintosh (1974; pp. 85–91; see also Gewirtz and Davis, 2000) identified two accounts of higher-order conditioning that have enjoyed an enduring appeal. One is closely aligned to conventional accounts of Pavlovian conditioning, wherein an association is held to form between the CS representation and either the US representation (i.e., a stimulus-stimulus association) or the processes responsible for responses that it generates (i.e., a stimulus-response association). For higher-order conditioning, it has been argued that an association forms between stimulus A and the US (or processes involved in generating the CR) through a process of representation mediated learning. Thus, for second-order conditioning, the X→US trials might allow A to become linked to the representation of the US that is retrieved by X on A→X trials (Konorski, 1948, p. 68) or to processes more directly responsible for the CR to X (Pavlov, 1927, p. 105; Rizley and Rescorla, 1972). Whereas for sensory preconditioning, the A→X trials might allow the representation of A retrieved by X on X→US trials to be linked to the US (e.g., Ward-Robinson and Hall, 1996, 1998; see also, Holland, 1981; Hall, 1996; Iordanova et al., 2011). Accounts based upon representation mediated learning are often contrasted with the simpler possibility that a (directional) associative chain underpins higher-order conditioning (e.g., Gewirtz and Davis, 2000). Here, X→US pairings allow an association to form between representations of X and the US, or those processes responsible for the UR, while A→X pairings enable an association to develop between representations of A and X. The efficacy of the associative chains, A→X→US or A→X→UR, will then determine the propensity for A to elicit conditioned responding. However, the accounts described above and depicted in Figure 1 are challenged by the conditions under which higher-order conditioning is observed and how it is evident in behavior.

## A Synthesis of Unresolved Issues

### The Conditions Under Which Higher-Order Conditioning Is Observed

When there is a trace interval between a CS and US (i.e., X→trace→US), conditioned responding during the CS is normally less evident than when there is no interval (see Mackintosh, 1983, pp. 86–89). The accounts of higher-order conditioning outlined above seem constrained to predict that when there is a trace interval between X and the US the CR to A should also be less marked: X→trace→US trials will be an ineffective basis for X to retrieve the US (or evoke the UR) on A→X trials in second-order conditioning procedures, and X→trace→US will be an ineffective vehicle for the retrieved representation of A to become linked to the US in sensory preconditioning procedures. Similarly, the final X→US or X→UR link in any associative chain will be less effective (in both procedures) after X→trace→US trials. However, trace conditioning with X enhances conditioned responding to A in both sensory preconditioning (Ward-Robinson and Hall, 1998; Lin and Honey, 2011; see also, Kamil, 1969) and second-order conditioning procedures (Lin and Honey, 2011; see also, Cole et al., 1995; Barnet and Miller, 1996). Another simple observation is similarly problematic: Extinguishing first-order conditioned responding to X, before test trials with A, does not (always) reduce the capacity of A to generate responding in sensory preconditioning (Ward-Robinson and Hall, 1996) or second-order conditioning procedures (e.g., Rizley and Rescorla, 1972; Cheatle and Rudy, 1978; Amiro and Bitterman, 1980; Nairne and Rescorla, 1981; Archer and Sjödén, 1982; but see Rescorla, 1982). These results are inconsistent with an associative chain account to the extent that the efficacy of the final link in the chain should have been reduced by extinguishing X, and they have been taken to support the view that A has an association with the US (or its UR) that is independent of the association of X with the US (or its UR). A final intriguing observation about sensory preconditioning is that when A is presented together with X during the test, the resulting AX compound provokes more conditioned responding than when X is either presented alone or with a control stimulus (e.g., Ward-Robinson et al., 2001; Lin et al., 2013). By default, and ignoring the results from the trace conditioning procedure, these results have been taken to support a retrieval mediated learning account since it supposes that A has a basis to elicit conditioning responding independently of X. However, these results could also reflect the fact that the directly activated representation of a stimulus (X), and its trace or retrieved representations (X*; see Lin and Honey, 2011, 2016; Lin et al., 2013) can be discriminated from one another, and enter into separate associations that affect performance in distinct ways (Lin and Honey, 2010). For example, enhanced higher-order conditioning with trace conditioning could reflect the fact that the representation of X that is retrieved by A is more similar to the representation of X that enters into association with the US during trace conditioning than during standard conditioning. Also, whether the extinction of X does or does not affect responding to A could be determined by the similarity of the representation of X retrieved by A during the test to the representation of X that was subject to extinction (see Rescorla, 1982). Later, we will develop a more formal analysis of this suggestion, which relies on representations of X, its trace and retrieved forms being dynamically coded in terms of the dimension of perceived intensity, and forming part of what is learned about a given stimulus.

### How Higher-Order Conditioning Is Evident in Behavior

Higher-order conditioning procedures include two types of trial, A→X and X→US, and there has been an understandable focus on how X→US trials enable responding to A. However, A→X trials can—in and of themselves—generate behavior. For example, when an auditory stimulus is paired with a localized visual stimulus (i.e., A→X), A comes to elicit an orienting response that reflects the location in which X is presented (e.g., Honey et al., 1998a,b; see also, Narbutovich and Podkopayev, 1936; cited in Konorski, 1948, p. 91; Silva et al., 2019). Any complete analysis of higher-order conditioning needs to address the fact that A will come to elicit behaviors that reflect the nature of both the US and X (see Lin and Honey, 2011, 2016; Lin et al., 2013). Not considering how the nature of the retrieved X might affect behavior to A is a pervasive issue with both informal accounts of higher-order conditioning and more formal models of Pavlovian conditioning: How do the proposed associative structures generate different forms of behavior? This process has been left underspecified by both formal models of Pavlovian conditioning (e.g., Mackintosh, 1975; Rescorla and Wagner, 1972; Pearce and Hall, 1980; Wagner, 1981) and informal accounts of higher-order conditioning.

The accounts of higher-order conditioning that we have considered assume that the associations responsible for performance are directional. For accounts based on representation mediated learning, the association is from A to the US (i.e., A→US), whereas for those based on an associative chain they are from A to X (i.e., A→X) and from X to the US (i.e., X→US). The requisite additional assumption is that performance is (ordinally) related to either the strength of the association between A and the US (i.e., V_A-US_), or the product of the links in the associative chain (i.e., V_A-X-US_ = V_A-X_ × V_X-US_; see Rescorla and Wagner, 1972). But, we know that accounts based on such assumptions are, at best, incomplete: The conditioned behavior generated by X→US trials reflects both the properties of the US and of the CS (e.g., Timberlake and Grant, 1975; see also, Holland, 1977; Patitucci et al., 2016; Iliescu et al., 2018). In fact, following Holland (1977, 1984), we can broadly distinguish between CS-oriented conditioned responding (e.g., sign-tracking; Hearst and Jenkins, 1974; see also, Davey and Cleland, 1982; Flagel et al., 2009) and US-oriented responding (e.g., goal-tracking; Boakes, 1977). Directional associations or chains of such associations from a CS to the US provide no foundation for CS-oriented conditioned behaviors^1^. Similarly, behaviors generated through Pavlovian conditioning (e.g., X→US) are not (quantitatively or qualitatively) the same as those generated by higher-order conditioning trials (e.g., A→X). This should be so if higher-order conditioned behavior is generated solely by associative activation of the US representation (see Holland and Rescorla, 1975; see Pavlov, 1927). Two examples from quite different preparations will suffice.

Stanhope (1992) gave hungry and thirsty pigeons training where keylight X was paired with food and keylight Y was independently paired with water. As a result, the pigeons directed pecks to X and Y, but those to X (the food keylight) were of greater force than those to Y (the water keylight; see Jenkins and Moore, 1973). The pigeons were then given trials where keylight A was paired with X while B was paired with Y. As a result, A and B came to elicit keypecking (see Rashotte et al., 1977), but the force of the keypecks to A and B did not differ in force (see also, e.g., Holland, 1977). Dwyer et al. (2012) gave thirsty rats separate access to two flavor compounds containing two flavors (A with X and B with Y); and then rats received access to X paired with illness and access to Y that was not. This procedure resulted in a reluctance to consume X relative to Y, and also A relative to B (see Rescorla and Cunningham, 1978). An important further finding was that while the first-order aversion was also evident in how rats consumed X (i.e., as a reduction in lick cluster size, indicative of a reduction in hedonic responses; see Dwyer, 2012), the second-order aversion to A was not. Neither a mediated A→US association nor an A→X→US associative chain provides a principled basis for the dissociations observed by Stanhope (1992) and by Dwyer et al. (2012; see also Holland and Rescorla, 1975).

## A More Formal Analysis

**Figure 2:**
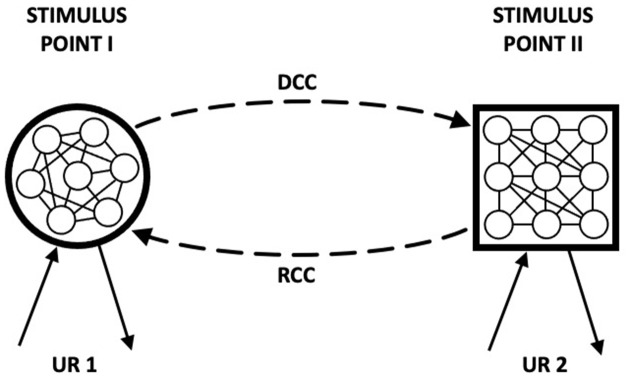
“Figure 8 Scheme of the conditioned reflex with bidirectional connection. DCC, direct conditioned connection; RCC, reverse conditioned connection; UR 1, unconditioned reflex No. 1; UR 2, unconditioned reflex No. 2.” Adapted from Asratian (1965). Note that while Stimulus Points I and II are reciprocally connected, the elements within Stimulus Points I and II are neither fully interconnected with one another and nor is their nature made explicit.

The model that we now describe builds on the assumption that learning involves the development of reciprocal associations: a central feature of the HeiDI model (see Honey et al., 2020a,b,c). This assumption provides a basis for the fact that conditioning can result in both an increase in CS-oriented and US-oriented behaviors to a CS, and was foreshadowed by Asratian (1965). Figure 2 is an adaptation of Figure 8 (Asratian, 1965; p. 179) where standard conditioning trials are held to result in a directly conditioned connection (DCC) and reverse conditioned connection (RCC) between Stimulus Points I and II (e.g., A and X, or X and the US). UR 2 can be generated both through direct activation of Stimulus Point II and through DCC by activation of Stimulus Point I, and UR 1 can be generated through activation of Stimulus Point 1 and by activation of Stimulus Point II through RCC. There is evidence to support the idea that reciprocal associations are formed during CS→US pairings (e.g., Asch and Ebenholtz, 1962; Heth, 1976; Tait and Saladin, 1986; Zentall et al., 1992; Gerolin and Matute, 1999; Arcediano et al., 2005).

**Figure 3:**
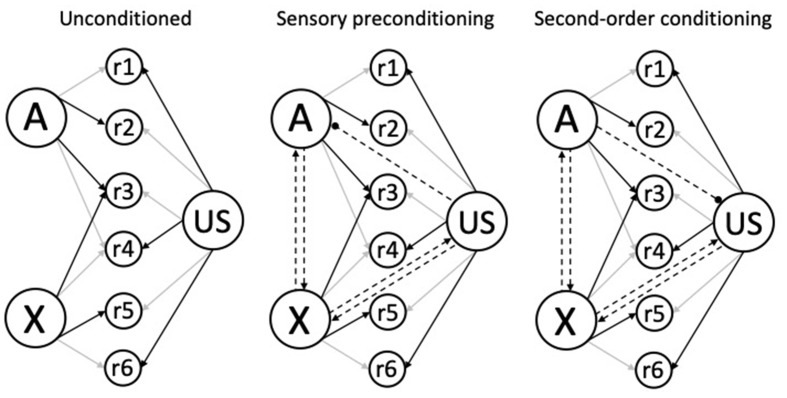
Schematic associative structures for higher-order (excitatory) conditioning. The left structure shows the unconditioned links from the CSs (A and X) and the US to response-generating units (r1-r6) before conditioning. The darkness of the arrows indicates link strength: A is strongly linked to r2 and r3, B is strongly linked to r3 and r5; and the US is strongly linked to r1, r4, and r6; and the remaining unconditioned links are weak or absent. The central and right structures show the reciprocal associations between the A and X, and between the X and US nodes (denoted by the dashed lines with arrowheads), acquired during higher-order trials (e.g., A→X and X→US); with a directional inhibitory US→A association for sensory preconditioning (center panel) and an inhibitory A→US association in second-order conditioning (right panel; denoted by the dashed line with the circular end; based upon one interpretation of inhibitory learning). Adapted from Honey and Dwyer (under review).

The model described here and developed in Honey and Dwyer (under review), has three components: (1) Learning rules together with the associative structures that they generate; (2) performance rules that determine how those structures generate different behaviors; and (3) a function that specifies the similarity between a CS, its trace, and retrieved forms, in terms of their perceived intensities. Schematics for the associative structures generated by higher-order conditioning trials (i.e., A→X and X→A) are depicted in Figure 3. We assume that the unconditioned structure has existing links of differing strengths from A, X, and the US to a set of response units (r1-r6; left panel), and that reciprocal (excitatory) links form between A and X, and between X and the US during both sensory preconditioning (middle panel) and second-order conditioning (right panel). In the case of sensory preconditioning conditioning, the X→US trials will also result in the formation of an accompanying inhibitory US→A link, whereas in the case of second-order conditioning, the A→X trials result in the formation of an inhibitory A→US link (see next paragraph).

In general terms, the formation of reciprocal links between the components of higher-order conditioning trials (A, X, and the US) provides a mechanism by which conditioned responding (to X) and higher-order conditioning (to A) are affected by the properties of the components of any given trial. In the case of higher-order conditioning, performance during A will reflect its properties (e.g., Holland, 1977; Patitucci et al., 2016; Iliescu et al., 2018), and those of the stimuli with which it is associated: X (Honey et al., 1998a,b; Silva et al., 2019; see also, Narbutovich and Podkopayev, 1936; cited in Konorski, 1948, p. 91) and the US (e.g., Holland and Rescorla, 1975; Holland, 1977; Stanhope, 1992; Dwyer et al., 2012). Similarly, performance to X will reflect the stimulus itself as well as its associations with A and the US. The issue then becomes one of specifying how the combined associative strengths within the extended associative structures (see Figure 3) is distributed to reflect the properties of A through the response units it is connected to and those of the retrieved representations of X and US. Following HeiDI (Honey et al., 2020a), we assume that they do so in proportion to their perceived intensities: for example, if the perceived intensity of A is higher than that of the retrieved memories of X or the US then a greater proportion of the combined associative strength would generate responses that are linked to A. Finally, we assume that this process is modulated by the similarity between the perceived intensities of the stimuli presented at the test (e.g., the associatively retrieved memory of X) to their perceived intensities on the conditioning trials (see Ward-Robinson and Hall, 1996, 1998; Ward-Robinson et al., 2001; Lin and Honey, 2011, 2016; Lin et al., 2013; see also, Kamil, 1969; see also, Cole et al., 1995; Barnet and Miller, 1996). We now give formal expression to these general ideas.

### Learning Rules

The formation of reciprocal associations between stimulus 1 and stimulus 2, having perceived intensities of α_1_ and α_2_, is determined by two equations: ΔV_1-2_ = α_1_(c.α_2_ − ΣV_TOTAL-2_); and ΔV_2-1_ = α_2_(c.α_1_ − ΣV_TOTAL-1_)^2^. These rules underpin the HeiDI model (Honey et al., 2020a). For both equations, associative changes on a given trial (ΔV_1-2_ and ΔV_2-1_) are influenced by pooled error terms (i.e., c.α_2_ − ΣV_TOTAL-2_ and c.α_1_ − ΣV_TOTAL-1_) in which ΣV_TOTAL-2_ and ΣV_TOTAL-1_ are the summed associative strengths of stimuli present on that trial to the subscripted stimulus (_1_ or _2_). The maximum possible associative strengths are given by c (which is 1 in units of V) multiplied by the perceived intensities of the stimuli (α_2_ and α_1_)^3^. Otherwise, the learning rules are simplified extensions to the one developed by Rescorla and Wagner (1972; see also, McLaren et al., 1989)^4^.

Equations 1 and 2 reference these generic equations to the critical A→X and X→A associations, and Equations 3 and 4 reference them to the X→US and US→X associations (analogous equations can be specified for the reciprocal links between A and the US). The maximum associative strength in Equation 3 is set by β_US,_ which is the learning rate parameter in Equation 4.


(1) $${\text{ΔV}}_{\text{A-X}}{\text{= α}}_{\text{A}}\text{(c}{\text{.α}}_{\text{X}}-\sum {\text{V}}_{\text{TOTAL-X}}\text{)}$$

(1) $${\text{ΔV}}_{\text{X-A}}{\text{= α}}_{\text{X}}\text{(c}{\text{.α}}_{\text{A}}-\sum {\text{V}}_{\text{TOTAL-A}}\text{)}$$

(1) $${\text{ΔV}}_{\text{X-US}}\text{= }{\text{α}}_{\text{X}}\text{(c}{\text{.β}}_{\text{US}}-\sum {\text{V}}_{\text{TOTAL-US}}\text{)}$$

(1) $${\text{ΔV}}_{\text{US-X}}\text{= }{\text{β}}_{\text{US}}\text{(c}{\text{.α}}_{\text{X}}-\sum {\text{V}}_{\text{TOTAL-X}}\text{)}$$

This analysis already affords additional explanatory power in the context of demonstrations of higher-order conditioning. For example, the analysis provides a simple explanation for (so-called) backward sensory preconditioning (Ward-Robinson and Hall, 1996, 1998). In this case, the fact that X→A pairings replace the typical A→X pairings has been taken to mean that an A→X→US chain cannot be constructed upon which to generate conditioned responding to A. The suggestion that X→A pairings enable reciprocal associations to form between X and A means that an A→X→US associative chain is generated. The same form of argument can be applied to the fact that when the usual X→US trials are replaced with US→X trials, subsequent presentations of A provoke marked (US-oriented) responding in a sensory preconditioning procedure (for an alternative analysis, see Miller and Barnet, 1993; see also, Cole and Miller, 1999). Finally, it has been demonstrated that second-order conditioning to A is reduced if the US is presented on the A→X trials (i.e., A→X→US; see Holland, 1980). This result is predicted to the extent that the US competes with A to become associated with X (because it is more intense; Mackintosh, 1976) and with X to become associated with A; and that this reduction in the strength of the A→X association outweighs the fact that X continues to be paired with the US.

### Performance Rules

Having specified the learning rules that generate the associative structures depicted in Figure 3, we now need to specify how these structures give rise to different conditioned behaviors. Our analysis is again based on HeiDI (Honey et al., 2020a,b,c). HeiDI separates the associative strengths of the CS→US and US→CS associations (Hebb, 1949) from the influence on performance of the intensities of the (presented) CS and (retrieved) US (see Hull, 1949). Thus, when the CS is presented the combined strength of the reciprocal associations [V_COMB_ = V_CS-US_ + (numerical value of V_CS-US_ × V_US-CS_)] is distributed into CS- and US-oriented components (R_CS_ and R_US_, respectively).^5^ With this distribution being determined by the perceived intensity of the CS (α_CS_) relative to the (retrieved) US (β_US_, as retrieved by the CS; see Holland, 1977; Patitucci et al., 2016). In general, this means that when α_CS_ is higher than β_US_, the CS-oriented component is greater than the US-oriented component, and when β_US_ is higher than α_CS_ the reverse is true. Individual differences in α_CS_ and β_US_ would be reflected in both CS-oriented and US-oriented responding and learning through the error-correcting learning rules. It is now time to consider how the extended associative structures depicted in Figure 3 and generated through Equations 1–4, affect behavior.

First, we should specify how the excitatory links in the middle and right panels of Figure 3 are integrated when either A or X is presented. When A is presented, we can assume that its associative influence (denoted V_CHAIN A-X-US_) is the product of the numerical value of V_A-X_ and V_COMB X-US_; where V_COMB X-US_ is calculated in the manner described in the context of combining the reciprocal associations between a CS and US. To capture the additional effect of the inhibitory link between A and the US (in the right-hand panel of Figure 3) the influence of V_COMB A-US_ needs to be added. V_COMB A-US_ has a negative value in second-order conditioning and a value of zero in sensory preconditioning (see the bracketed terms in Equations 5–7). In contrast, should X be presented, V_COMB X-US_ would be combined with the V_CHAIN_ _X-A-US_.

Now, these combined values can be separated into three components that influence the links from A, X, and the US to r1-r6 in proportion to their (perceived) intensities (see Equations 5–7). Upon presentation of A at test, its intensity would be directly given (i.e., by α_A_; unless one was assessing test performance during its trace; see Lin et al., 2013); while that of the (retrieved) X would be given by the absolute numerical value of V_A-X_ (for sensory preconditioning), and the sum of the absolute numerical values of V_A-X_ and V_A-US-X_ (for second-order conditioning). This allows the perceived intensity of a retrieved stimulus to exceed its α value, in much the same way as the Rescorla-Wagner model (see Kremer, 1978). β_US_ would be given by the absolute numerical value of V_A-X-US_ for sensory preconditioning, while for second-order conditioning it would be given by the absolute numerical value of the sum of V_A-X-US_ + V_A-US_. The fact that the link from A to the US is indirect and weak, in contrast to the direct link between X and the US, will result in a greater bias toward CS-oriented (R_A_) than US-oriented (R_US_) behaviors during A than during X (see Dwyer et al., 2012; Holland and Rescorla, 1975; Stanhope, 1992).


(1) $${\text{R}}_{\text{A}}\text{=}\frac{{\text{α}}_{\text{A}}}{{\text{α}}_{\text{A}}{\text{+ α}}_{\text{X}}{\text{+ β}}_{\text{US}}}\left({\text{V}}_{\text{CHAIN A-X-US}}{\text{+ V}}_{\text{COMB A-US}}\right)$$

(1) $${\text{R}}_{\text{X}}\text{=}\frac{{\text{α}}_{\text{X}}}{{\text{α}}_{\text{A}}{\text{+ α}}_{\text{X}}{\text{+ β}}_{\text{US}}}\left({\text{V}}_{\text{CHAIN A-X-US}}{\text{+ V}}_{\text{COMB A-US}}\right)$$

(1) $${\text{R}}_{\text{US}}\text{=}\frac{{\text{β}}_{\text{US}}}{{\text{α}}_{\text{A}}{\text{+ α}}_{\text{X}}{\text{+ β}}_{\text{US}}}\left({\text{V}}_{\text{CHAIN A-X-US}}{\text{+ V}}_{\text{COMB A-US}}\right)$$

**Figure 4:**
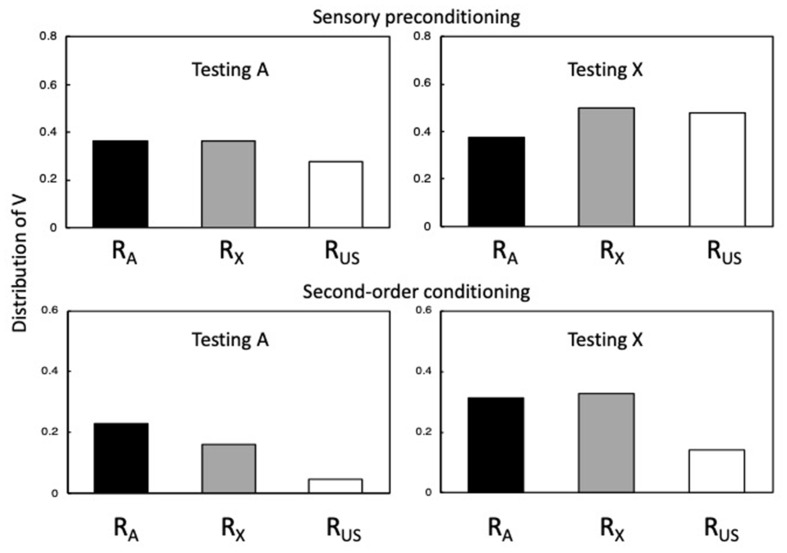
Simulations of sensory preconditioning and second-order conditioning. The output values for R_A_, R_X,_ and R_US_ were generated for A and X using Equations 1–7 with α_A_ = α_X_ = β_US_ = 0.80. There were 10 A→X trials and 2 X→US trials for the sensory preconditioning simulation, and 10 X→US trials and 2 A→X for the second-order conditioning simulation. For both simulations, the values of a R_A_, R_X,_ and R_US_ were then computed for A. Adapted from Honey and Dwyer (under review).

The influence of R_A_, R_X,_ and R_US_ on the response-generating units (r1-r6 in Figure 3) will reflect the strengths of the unconditioned links between A, X and the US and r1-r6; for example, through multiplying R_A_, R_X,_ and R_US_ by the weights from A, X and the US to r1-r6 (see Honey et al., 2020a). Figure 4 presents some indicative simulations of the values of R_A_, R_X,_ and R_US_.

The upper panels of Figure 4 depict simulations of sensory preconditioning, while its lower panels depict simulations of second-order conditioning. In both cases, α_A_ = α_X_ = β_US_ = 0.80. The left-hand panels show the values of R_A_, R_X,_ and R_US_ for the presentation of A, which were calculated after 10 A→X trials and 2 X→US trials (sensory preconditioning) and after 10 X→US trials and 2 A→X trials (second-order conditioning). The right-hand panels show the corresponding values for the presentation of X. Values that are positive indicate the presence of higher-order conditioning. In the upper left panel, R_A_ and R_X_ output values are positive and similar, with both being higher than R_US_. The similar output values for R_A_ and R_X_ reflect that they have the same α value and V_A-X_ (the numerator in Equation 6) ≈ α_X_ because it has approached asymptote over the course of 10 A→X trials. R_US_ has a lower value since the numerator in Equation 7 derives from the (absolute) numerical value of V_A-X_ × V_X-US_; which aligns to the perceived intensity of the US as retrieved by A through X. The upper right-hand panel shows the corresponding values for X^6^.

R_A_ is lower than R_X_ and R_US_ because the value of V_X-A_ declines over the course of X→US pairings. These simulations reveal that while R_A_ and R_X_ (aligned to CS-oriented responding) are similar whether A or X is tested, R_US_ (aligned to US-oriented responding) takes a higher value during X than A.

The lower panels of Figure 4 show output values for simulations of second-order conditioning, generated with the same parameters as sensory preconditioning, and after the same number of trials in the first and second stages (10 X→US trials and 2 A→X^7^). Comparing first the upper and lower panels (noting their different scales), R_A_ and R_X_ output values were relatively similar during A (and X) for simulations of sensory preconditioning and second-order conditioning (see Barnet et al., 1991). However, R_US_ values were far lower for second-order conditioning than for sensory preconditioning. Indeed, if α_A_ and α_X_ are set to lower values, it results in the components of the excitatory chain becoming less effective with the consequence that there is now no second-order conditioning. In any case, the fact that R_US_ is particularly low for second-order conditioning (relative to R_A_ and R_X_) reflects the influence of the inhibitory V_A-US_ on the calculated value of β_US_: When A is tested, the value of β_US_ = numerical values of V_A-US_ (inhibitory) + V_A-X_ × V_X-US_ (excitatory); and when X is tested, β_US_ = numerical values of V_X-A_ × V_A-US_ (inhibitory) + V_X-US_ (excitatory). A further difference from sensory preconditioning is that during the test with A the output value for R_A_ is greater than for R_X_. This difference derives from the fact that in sensory preconditioning V_A-X_ (the numerator in Equation 6) ≈ α_X_, whereas in second-order conditioning V_A-X_ does not reach asymptote as a consequence of two A→X trials, and is further constrained by V_A-US-X_ being negative. The simulations in Figure 4 can be aligned with results reported by Stanhope (1992) using an autoshaping procedure in pigeons, and Dwyer et al. (2012) using a flavor-aversion procedure in rats: If pecking a keylight (in pigeons) and fluid consumption (in rats) is equated to CS-oriented responding (generated by R_A_ and R_X_), and the force of pecks and lick cluster size is equated with US-oriented responding (generated by R_US_).

### Similarity Function

The central idea captured in Equations 5–7 is that the relative intensities of components of the test pattern (some present and others retrieved) determine how the associative structures depicted in Figure 3 generate behaviors aligned to those components (A, X, and US). What they do not capture is how differences in the intensities of a given component between test and conditioning influences R_A_, R_X_, and R_US_. In Equations 5–7 identity is simply assumed. There are three reasons why this needs to be addressed: First, Equations 5–7 have no (internal) mechanism for restricting conditioned behavior to stimuli that have been present on conditioning trials or to those associated with them: Associatively neutral stimuli might well influence the distribution of associative strength, but without necessarily eliciting anything other than unconditioned responses (see Pavlov, 1927, p. 44; see also, Honey et al., 2020a). Second, animals can learn discriminations in which the effective stimuli involve: (a) whether the same stimulus is presented at one intensity or a different intensity (e.g., Inman et al., 2016; for a review, see Inman and Pearce, 2018), and (b) whether the same stimulus has been presented more or less recently (e.g., Lin and Honey, 2010; see also, Pavlov, 1927; Mackintosh, 1974, p. 104; Staddon and Higa, 1999; Staddon, 2005). The latter observation reducing to the former once different components of a decaying trace are equated with different stimulus intensities; both observations suggest that different intensities of a given stimulus can enter into different associations, but also that there is generalization between those intensities. Third, the idea that the representation of the CS includes the intensity at which it is presented affords an account for when higher-order conditioning is observed: As already noted, trace conditioning might enhance higher-order conditioning because when A retrieves X at test (i.e., X*) it is more similar in perceived intensity to the stimulus that became linked to the US during trace conditioning (X*) than standard conditioning (X; Ward-Robinson and Hall, 1998; Lin and Honey, 2011; see also, Kamil, 1969; Cole et al., 1995; Barnet and Miller, 1996). It would also help to explain the fact that higher-order conditioning to A can be left unaffected by the extinction of responding to X (e.g., Rizley and Rescorla, 1972; Cheatle and Rudy, 1978; Amiro and Bitterman, 1980; Nairne and Rescorla, 1981; Archer and Sjödén, 1982; Ward-Robinson and Hall, 1996; but see, Rescorla, 1982): Because X (rather than the trace, X*) would undergo extinction when X is presented (see Kamin, 1969; Mackintosh, 1976). Finally, when A is presented with X at test, A will retrieve X*, which has strength independently of X itself (e.g., Ward-Robinson et al., 2001; Lin et al., 2013). This analysis is plausible, but without a function that specifies the similarity between the perceived intensities of stimuli, their traces, and retrieved representations it remains tendentious (see Lin and Honey, 2011, 2016; see also, Lin et al., 2013). However, one such function is presented below in the context of how the retrieved memory of X affects performance during the presentation of A (i.e., in a modification of Equation 6).


(1) $${\text{R}}_{\text{X}}\text{=}\frac{{\text{α}}_{\text{X}}}{{\text{α}}_{\text{A}}{\text{+ α}}_{\text{X}}{\text{+ β}}_{\text{US}}}\left(\left({\text{α}}_{\text{X-R}}{\text{Sα}}_{{}_{\text{X-C}}}{\text{×V}}_{\text{CHAIN A-X-US}}\right){\text{+ V}}_{\text{COMB A-US}}\right)$$

Where:

α_X_ = α_X-R_ = |$\frac{1}{C}$V_A-X_| and α_X-C_ = α of X upon delivery of the US


$${\text{α}}_{\text{X-R}}{\text{Sα}}_{\text{X-C}}\text{=}\frac{{\text{α}}_{\text{X-R}}}{\left({\text{α}}_{\text{X-R}}\text{+}\left|{\text{α}}_{\text{X-C}}-{\text{α}}_{\text{X-R}}\right|\right)}\text{×}\frac{{\text{α}}_{\text{X-C}}}{\left({\text{α}}_{\text{X-C}}\text{+}\left|{\text{α}}_{\text{X-C}}-{\text{α}}_{\text{X-R}}\right|\right)}$$

The function (α_X-R_Sα_X-C_) introduced in Equation 8 (in the gray boxes) determines the similarity (S) of two values: The numerical value of V_A-X_ (denoted α_X-R_) and its conditioned counterpart or trace (denoted α_X-C_). It is worth remembering that when V_A-X_ reaches asymptote, its numerical value ≈ α_X_, which means that α_X-R_ ≈ α_X-C_. This function is also applied to modify the bracketed term in Equations 5 and 7 when A is presented. Its basic properties are simple: When the values of α_X-R_ and α_X-C_ are close together then α_X-R_Sα_X-C_ approaches 1, but as they diverge then α_X-R_Sα_X-C_ approaches 0. Applying these ideas to how α_X-R_ affects performance is also simple. Because the asymptote for V_A-X_ during A→X training is α_X_, when A is presented at test α_X-R_ will have approached α_X_ over the A→X trials. If A→X training had proceeded until V_A-X_ reached asymptote then α_X-R_ and α_X-C_ would be maximally similar, provided α_X_ during X→US conditioning trials was the same as during A→X trials (as it usually is). Now, we can appreciate how α_X-R_Sα_X-C_ varies when α_X_ has one value for A→X trials (e.g., 0.50) and is then reduced for X→US trials (e.g., 0.45); this reduction in α_X-C_ is intended to mimic the effect of introducing a trace interval between X and the US (see Lin and Honey, 2011, 2016; Lin et al., 2013). It should be clear that before V_A-X_ has reached asymptote during A→X trials, its numerical value can match more closely 0.45 than 0.50; and that as V_A-X_ tends to 0.50 for A→X trials the numerical value of V_A-X_ will become closer to 0.50 than 0.45. The accuracy of this analysis was confirmed by simulations.

**Figure 5:**
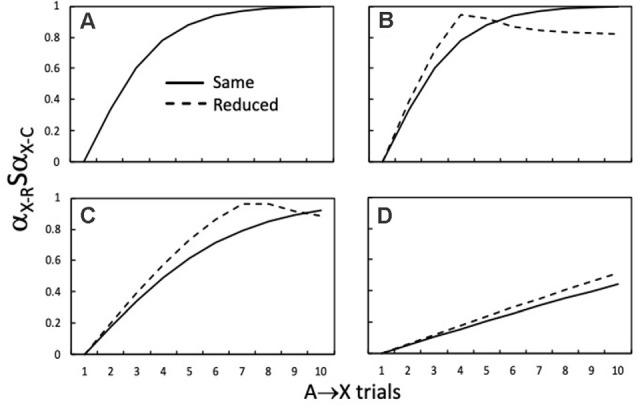
How the similarity *(*α_X-R_Sα_X-C_*)* of the retrieved X *(*α_X-R_) to the conditioned X *(*α_X-C_) during a test with A varies with the number of initial A→X trials. The continuous lines denote α_X-R_Sα_X-C_ output values when the α_X_ value (0.50) used to compute changes in V_A-X_ (i.e., α_X-R_) was the *same* as that for α_X-C_ on X→US conditioning trials. α_A_ was 0.50 in panels **(A,B)**, 0.30 in panel **(C)**, and 0.10 in panel **(D)**. The dashed lines denote α_X-R_Sα_X-C_ output values when the α_X_ value used to compute changes in V_A-X_ (0.50; i.e., α_X-R_) was *reduced* to 0.45 for α_X-C_ to calculate α_X-R_Sα_X-C._ This manipulation is akin to using trace conditioning for X→US trials. Adapted from Honey and Dwyer (under review).

Figure 5 shows how α_X-R_Sα_X-C_ varies as a function of the number of A→X training trials during the first stage of training. The continuous lines show α_X-R_Sα_X-C_ when α_X-R_ and α_X-C_ are generated by the same α_X_ value (e.g., 0.50), as in standard higher-order conditioning procedures. Comparison of the continuous lines across Figures 5A–D shows that the rate at which maximum similarity is approached, across a series of A→X trials, decreases as α_A_ is reduced from 0.50 (Figures 5A,B), to 0.30 (Figure 5C), and then 0.10 (Figure 5D). Turning now to the dashed lines in Figures 5B–D, it is clear that there is a period of initial A→X training when reductions in α_X-C_ increase α_X-R_Sα_X-C_ compared to when α_X-C_ is the same (i.e., 0.50 for the continuous lines). With more extended A→X training this pattern reverses as α_X-R_ (i.e., V_A-X_) approaches 0.50 and consequently deviates from the reduced value of α_X-C_ (i.e., 0.45). This reversal is apparent in Figures 5B,C, but not within 10 trials in Figure 5D.

The take-home message from these simulations is that trace conditioning will have the potential to enhance higher-order conditioning if A is tested when A→X training has left V_A-X_ within the range where the dashed line has higher values than the continuous line. The influence of such increases in similarity on higher-order conditioning will be contingent on them more than counteracting any direct effect of reducing α_X-C_ on the efficacy of the X-US component of the chain (i.e., V_CHAIN A-X-US_). In fact, simulations reveal that increases in R_A_, R_X_, and R_US_ of between 10% to 20% are produced by reducing α_X-C_ by 10%, which is in the range where reducing α_X-C_ has little effect on the rate at which V_X-US_ approaches the asymptote determined by β_US._ These effects of similarity are more marked for sensory preconditioning than second-order conditioning (see Honey and Dwyer, under review).

Our formal analysis assumes that the value of α on a conditioning trial is encoded and is one basis for generalization between a CS presented at one intensity and the same CS but delivered at a different intensity. It also assumes that there is a (computational) equivalence between different α (and β) values generated by changing a stimulus physically (e.g., Inman et al., 2016) and the values generated through the central processes of decay and retrieval (e.g., Lin and Honey, 2010; see also Iliescu et al., 2020). In addition to providing an analysis for how trace conditioning can enhance higher-order conditioning, it can also explain related observations: the facts that extinction of X is not always reflected in responding to A, and the compound AX generates more responding than X in sensory preconditioning procedures. As already noted, the effects of extinction treatments involving the presentation of X will be more likely to impact its directly activated α value as opposed to its decaying value through a process of overshadowing (Mackintosh, 1976); and whether this affects higher-order conditioning will depend on whether the representation of X that supports responding to A (which is determined by the strength of the A→X association; see Rescorla, 1982) is similar to its directly activated or decaying forms. Equation 8 provides a formal example of how test performance is affected by the similarity between the value of X retrieved by A as a consequence of A→X trials and its encoded value during conditioning trials. According to our analysis, AX will generate more responding than X because the associative chain can exert an independent influence on the US representation (for further details, see Honey and Dwyer, under review).

To close the theoretical loop, the learning rules (e.g., ΔV_1-2_ = α_1_(c.α_2_ − ΣV_S-TOTAL-2_)) can be modified to reflect the fact that the associative strengths of stimuli contributing to ΣV_S-TOTAL-2_ (including V_1-2_) need to be scaled by their similarity (subscript s) to their intensities when conditioned (see Pearce, 1994). For instance, Equation 3 can be re-cast as Equation 9, where the subscript s denotes this scaling process. The similarity function is as before, but α_X-R_ is the perceived intensity of the CS on previous trials, while α_X-C_ = α_X_ of the same CS on the current trial. In this way, the perceived intensity of a CS is encoded as one component of what is learned on a conditioning trial (if α_X_ changes from one trial to the next then new learning occurs), which reflects the generalization of associative strength between a stimulus conditioned at one intensity and later presented at another intensity (i.e., ΣV_S-TOTAL-US_ is reduced because α_X-R_Sα_X-C_ < 1). It should be recognized, however, that increases and reductions in intensity have different effects on behavior through the proportion terms in the equations that determine the distribution of associative strength (e.g., in Equation 8). Finally, it is worth noting that the effect of changing α_X_ from one trial to the next on the US→X association will be that V_US-X_ homes in on the new α_X_ (see Equation 4), which parallels the fact that changes in US intensity across trials affects the asymptote of the X-US association.

## Discussion: Some Concluding Considerations

Understanding higher-order conditioning has theoretical and translational value, but traditional (informal) accounts of this phenomenon are poorly equipped to address two fundamental issues: What is learned and how it is expressed. The analysis described here and developed in Honey and Dwyer (under review) borrows from HeiDI, which is a model of Pavlovian learning and performance (Honey et al., 2020a). The learning and performance rules are derived from HeiDI, but their influence is modulated by a similarity function. This function specifies the similarity between the same nominal stimulus, which can take different perceived intensities as a result of manipulating the intensity at which it is delivered and through processes of retrieval or trace decay. The resulting analysis has clear implications for behavioral neuroscience, where group-level differences in higher-order conditioning should be interpreted with caution: Changes in a given behavioral measure of higher-order conditioning consequent on a manipulation might have a variety of origins. For example, differences in learning or performance might not reflect differences in the underlying learning mechanisms but rather changes to: α (for A and X), β (for the US), or their associated decay functions (see Honey and Good, 2000); or indeed the requisite (neural) computations involving the processes represented by these parameters.

In developing this more formal analysis of higher-order conditioning, no appeal has been made to any process of retrieval mediated learning or stimulus-response learning. This is not intended to suggest that such forms of learning are without consequence, but simply that they are not required by the available evidence. For example, the model presented here could accommodate retrieval mediated learning between A and the US in a sensory preconditioning procedure by substituting the numerical value of ΣV_TOTAL-A_ for α_A_: ΔV_A-US_ = 1/c.ΣV_TOTAL-A_(c.β_US_ − ΣV_S-TOTAL-US_); recall that multiplying ΣV_TOTAL-A_ by 1/c transforms it into a dimensionless scalar like α_A_. In this way, a retrieved stimulus, or stimulus trace, might acquire associative strength while limiting that acquired by other stimuli present on a conditioning trial. As we have noted, retrieved stimuli will also affect performance through the proportion terms in Equations 5–8 (see Holland, 1983). This analysis joins others that have attempted to provide a more specific account of the process of retrieval mediated learning, albeit that they do not apply as readily to higher-order conditioning as they do to other phenomena (e.g., Van Hamme and Wasserman, 1994; Dickinson and Burke, 1996; see also, Dwyer et al., 1998).

We should briefly comment on the complexity of the model. While the model has three components (relating to learning, performance, and similarity) it only has two free parameters: α (for A and X) and β (for the US); and their associated decay functions. It can also be summarized in two simple statements: 1. The perceived intensities of stimuli present during a test affect how learning represented within an extended associative structure affects performance; and 2. The similarity of the perceived intensities of the tested stimuli to conditioned stimuli within that structure modulates the translation of learning into performance.

Our use of the term *perceived intensity* clearly affords a potential analysis of individual differences in both Pavlovian conditioning and higher-order conditioning at the level of learning and performance (see Honey et al., 2020a,b,c), but also now in terms of the similarity between directly activated representations, their decaying traces, and retrieved forms. Pavlov (1927; p. 105) noted that there were marked individual differences in the strength of second-order reflexes: “*Among the experimental dogs one finds special types of nervous systems; in particular there are dogs with weak nervous systems in which this phenomenon is clearly expressed*.” The fact that there are significant individual differences in how learning is evident in behavior has been neglected by general-process models of learning. The model upon which our analysis is based, HeiDI, represents a prosaic approach to accommodating both quantitative and qualitative individual differences in conditioned behavior.

## Author Contributions

RH and DD contributed to the ideas presented in this article and to its preparation for publication. Both authors contributed to the article and approved the submitted version.

## Conflict of Interest

The authors declare that the research was conducted in the absence of any commercial or financial relationships that could be construed as a potential conflict of interest.

## Publisher’s Note

All claims expressed in this article are solely those of the authors and do not necessarily represent those of their affiliated organizations, or those of the publisher, the editors and the reviewers. Any product that may be evaluated in this article, or claim that may be made by its manufacturer, is not guaranteed or endorsed by the publisher.

## Footnotes

^1^Some combinations of stimuli might activate response units that generate behaviors that do not closely resemble those observed when the same stimuli are presented individually (e.g., conditioned freezing).

^2^The constant (c = 1 in units of V) is required to balance the equations in terms of the dimensions/units involved (see Honey et al., 2020a).

^3^The fact that the asymptotes and the rates at which they are reached are determined by α_1_ and α_2_ creates computational advantages when specifying the similarity of (1) the retrieved values of α_2_ and α_1_ (given by the numerical values of V_1-2_ and V_2-1_, respectively), and (2) their conditioned values α_2_ and α_1_.

^4^The rules have no independent lambda (λ) parameter to determine the asymptote for the V_1-2_ association (or for the V_2-1_ association). There is also no need to have separate learning rate parameters for when the target for the association (1 or 2) is present (e.g., β_E_) and absent (e.g., β_I_; see Honey et al., 2020b). β_I_ was required by the Rescorla-Wagner model − Δ *V*_CS-US_ = αβ(λ−Σ*V*) — to enable learning to occur when the US was absent and β would otherwise = 0.

^5^The reciprocal associations are combined in this way, rather than being simply mapped onto CS-oriented (US→CS) and US-oriented (CS→US) responding, to reflect the interactive nature of the reciprocal associations, but also to avoid the prediction that extinction of the CS would leave CS-oriented responding unaffected because it would only impact the CS→US association (see Iliescu et al., 2020).

^6^Here, α_A_ = the (absolute) numerical value of V_X-A_ (i.e., 1/c|V_X-A_|), α_X_ = α_X_ and β_US_ = the (absolute) numerical value of V_X-US_.

^7^Maintaining the number of trials of the two types (10 A→X and 2 X→US), rather than the number of trials in the two stages (10 for stage 1 and 2 for stage 2), results in extinction of the X→US association over the course of the 10 A→X trials.
